# Early-detection scheme based on sequential tests for low-latency communications

**DOI:** 10.1186/s13638-023-02240-9

**Published:** 2023-03-21

**Authors:** Diego Barragán-Guerrero, Minh Au, Ghyslain Gagnon, François Gagnon, Pascal Giard

**Affiliations:** 1 Department of Electrical Engineering, École de technologie supérieure, Montréal, H3C 1K3 QC Canada; 2 Departamento de Ciencias de la Computación y Electrónica, Universidad Técnica Particular de Loja (UTPL), Loja, 1101608 Ecuador; 3 Hydro-Québec’s Research Institute (IREQ), Varennes, J3X 1S1 Canada

**Keywords:** Early detection, Finite blocklength, Low latency communications, Multialternative sequential tests

## Abstract

We propose an early-detection scheme to reduce communications latency based on sequential tests under finite blocklength regime for a fixed-rate transmission without any feedback channel. The proposed scheme processes observations sequentially to decide in favor of one of the candidate symbols. Such a process stops as soon as a decision rule is satisfied or waits for more samples under a given accuracy. We first provide the optimal achievable latency in additive white Gaussian noise channels for every channel code given a probability of block error. For example, for a rate $R = 0.5$ and a blocklength of 500 symbols, we show that only $63\%$ of the symbol time is needed to reach an error rate equal to $10^{-5}$. Then, we prove that if short messages can be transmitted in parallel Gaussian channels via a multi-carrier modulation, there exists an optimal low-latency strategy for every code. Next, we show how early detection can be effective with band-limited orthogonal frequency-division multiplexing signals while maintaining a given spectral efficiency by random coding or pre-coding random matrices. Finally, we show how the proposed early-detection scheme is effective in multi-hop systems.

## Introduction

In fifth-generation cellular network technology (5 G), a large set of autonomous devices will require quick and reliable radio access. 5 G will enable scenarios like Internet-of-Things (IoT), where one cell is expected to contain up to 10$^5$ connected nodes; Gigabit wireless connectivity, with a download-speed target of 10 Gb/s; and Tactile Internet, with a end-to-end latency constraint of 1 ms and reliabilities near 10$^{-9}$ [1]. Several approaches have been proposed to tackle this new challenge, such as enhanced mobile broadband (eMBB), massive machine-type communication (mMTC) and ultra-reliable and low-latency communications (URLLC) [2].

URLLC focuses on technologies that require special attention in the time needed to transmit a packet under predefined reliability. Among these low-latency emerging technologies are applications like autonomous vehicles, interactive multi-player gaming, health remote surgery, real-time teleoperation, and virtual/augmented reality [3, 4].

The transition time of information in a communication system is the sum of all latencies inherent to the system. Some latency-source examples are the routing delay, the over-the-air transmission time, the network queuing, the number of active users, and many others. Latency sources are numerous and vary in nature.

With the aim of reducing the system latency, several approaches have been explored. At the physical layer alone, for example, researchers investigated more efficient channel codes, faster signal processing, new frame structures, and improved radio-resource management [5, 6].

Concerning channel coding, to achieve the latency and reliability goals in URLLC, the use of short blocklengths, also referred to as short blocklength regime, is mandatory [7]. However, there are some performance considerations with respect to capacity when the blocklength no longer tends to infinity. To characterize the penalty incurred in the rate under the finite blocklength (FBL) regime, Polyanskiy et al. [8] have developed a model to approximate the maximum code rate for several channels and power constraints. Such a model is called a normal approximation.

Along with short packets, we can reduce latency through a quick signal processing stage, i.e., have the receiver achieve a reliable decision using only a fraction of the transmitted-symbol length. A sequential probability test can reach such a decision [9–11]. However, for a large information block size, the receiver based upon a sequential test needs a significant number of tests to select which message was transmitted [12]. Interestingly, a list decoder before the sequential test can significantly reduce the number of candidate symbols by providing a list of the most probable messages, rendering the sequential detector feasible.

In this paper, focusing on fixed-rate communication without any feedback channel, we aim to reduce the detection latency by using a scheme that decodes a short packet with high reliability from fractions of the received signal.^1^ Such a scheme is referred to as an early-detection scheme (EDS), which comprises a list decoder and a sequential test to perform quick detection as soon as the probability of a reliable decision is high enough.

In the proposed scheme, we define the detection latency as the interval between the beginning of the symbol transmission and the moment of its correct detection [13].

This work contributes to the field of URLLC by combining sequential detection and short packet communication. The main contributions of this paper are summarized as follows:

- We provide detailed results about the maximal code size achievable under normal approximation in terms of physical variables like latency, power, and symbol duration.
- We provide analyses of the normalized average latency for various rates, blocklength, and block error rate (BLER) of practical interest.
- We conjecture a threshold for the sequential test when the receiver uses a list decoder to reduce the number of candidate messages.
- We provide the upper bound of the block error rate as a function of any given threshold when the system uses the EDS.
- We discuss how the proposed EDS is effective with orthogonal frequencydivision multiplexing (OFDM) signals via random coding or pre-coding Hadamard random matrices.
- We show that, in multi-hop links, the proposed EDS can reliably detect symbols before the end of their transmission, significantly reducing the latency while maintaining the error probability.

The remainder of this work is organized as follows. In Sect. 2, we present a background on short-packet communications and sequential probability-ratio tests. Some assumptions and comparison metrics are introduced in Sect. 3. In Sect. 3.1, we formulate the minimal-latency problem to investigate the optimal achievable latency. In Sect. 3.2, we define the EDS that can be employed for such a purpose. In Sect. 3.3, examples employing this proposed fastest-detection scheme are presented. In Sect. 4, results for the proposed scheme are discussed. Finally, Sect. 5 summarizes our main findings.

## Background

To guarantee reliable communication, Shannon established as a necessary condition the use of large blocklengths, a condition known as the infinite blocklength (IBL) regime [14]. However, in URLLC, it is necessary to use short blocklengths because the network delay is affected by the packet size. As the blocklength does not tend to infinity, the coding scheme is denominated the finite blocklength (FBL) regime. To determine the transmission conditions under FBL regime, Polyanskiy et al. [8] derived a framework to approximate both the achievability and converse bounds on the size of a code and the performance gap from the Shannon capacity. Hence, the maximum code rate $R^*$ is expressed as a function of the block error rate (BLER) $\epsilon$ and the finite blocklength *n*. In other words, the maximum code rate is the largest rate at which an encoder-decoder pair operates with a finite blocklength *n* and whose BLER is not greater than a predefined value $\epsilon$.

In this paper, we leverage the Polyanskiy’s bounds to construct a sequential hypothesis test that will be used to make an early decision on the received symbol. A sequential test processes the received samples as they periodically arrive at the detector based on the statistical distribution of the data source. These types of tests are characterized by the average number of samples used to make a decision and the probability of error in making that decision [11]. The first to propose such a test was Wald [15], who designed a sequential test for binary hypotheses called the sequential probability-ratio test (SPRT). The SPRT processes an independent and identically distributed (i.i.d.) random sequence and decides in favor of one hypothesis if the likelihood ratio between two candidate hypotheses exceeds a predefined threshold. The SPRT has proven to be asymptotically optimal for two hypotheses, i.e., it uses the minimum expected sample size compared with other tests. Besides, the SPRT is generalized in applications where more than two hypotheses need to be tested, like detecting a target in a multiple-resolution radar, serial acquisition of direct-sequence spread-spectrum signals, or quick fault detection and identification. Such a scheme is called a multihypothesis sequential probability-ratio test (MSPRT) [10].

The MSPRT processes observations sequentially to decide in favor of one of the *M* multiple hypotheses, where the test stops as soon as a decision rule is satisfied or waits for more samples under a given accuracy [9]. As stated by [10], there are two approaches to design a MSPRT: either find an optimal test, a complex and non-practical procedure; or extend a binary SPRT to the multi-hypothesis case based on recursive pairwise uses of the SPRT. Through an extended version of the SPRT, Dragalin et al. [9] proposed a couple of sequential tests of multiple hypotheses inspired by the Bayesian optimality arguments and a generalized likelihood ratio test. Such a test decides in favor of one hypothesis if some statistics reach a threshold.

Unlike [9] and [10], instead of considering the total number of messages *M* to determine the threshold, in this work, we will conjecture a threshold that accounts for the size of the list decoder, which is less than *M*. In other words, the test threshold depends on both the number of most probable candidate symbols as well as the targeted error probability.

There is a trade-off to be found between low latency and high reliability [4]. Achieving low latency requires sacrificing some reliability. For example, to guarantee high reliability, the system needs a retransmission technique, where once the receiver decides on a received message, it uses the noiseless feedback link to inform the transmitter that the next message can be sent [16]. Through this feedback channel, the system guarantees high reliability of communication at the cost of a drastically increased latency [17]. Therefore, avoiding the use of feedback will greatly reduce the latency at the cost of a sacrifice in reliability.

Therefore, unlike [18, 19], with the aim of minimizing latency, we avoid a feedback channel in this work since the latency would increase due to retransmission [4]. In addition, we use as a coding scheme a model based on the FBL regime, which determines the block size, code rate, and error probability of the proposed EDS. Thus, we combine short channel symbols with sequential detection to achieve a significant latency reduction in a communication system.

## Methods

As mentioned above, the EDS tries to reduce the detection latency by using a technique that decodes a short message with high reliability from fractions of the received packet. In order to achieve it, the proposed scheme combines a sequential test guided by list decoding.

The performance comparison will be provided in terms of the normalized average latency, which is a ratio between the average early-detection time and the symbol duration. We assume that the channel remains constant within the transmission time of a symbol, meaning that the symbol duration does not exceed the channel coherence time. This assumption means that if the coherence time of the channel is greater than the symbol duration, then the channel conditions will remain constant during the transmission of a single symbol. As a result, the transmission time of a symbol, which is determined by the symbol rate and the channel’s bandwidth, will be equal to the symbol duration.

The transmission mode chosen is a multi-carrier system. The symbols of a message are BPSK modulated and transmitted in parallel through the channel and that detection is performed simultaneously on the receiver. We chose BPSK modulation since it is simple to implement, and it is the signaling scheme that maximizes the distance between symbols, providing the maximum immunity to noise [20]. Furthermore, this selection is justified because our system does not consider feedback channels, and BPSK contributes to maintaining the required reliability.

Also, the EDS operates in a BLER range where the normal approximation is accurate [21]. In addition, our scheme imposes a commonly-used average-power constraint of the codewords, i.e., it includes the transmitted power restriction to model the devices’ battery capacity and regulatory constraints correctly [22].

### On the optimal achievable latency for synchronous detection schemes

#### Minimal achievable latency in the infinite-blocklength regime

In this section, we use an extension of the classical equation of channel capacity to formulate the parameters involved in the minimal latency problem.

A channel coding scheme adds redundancy to the user message to decrease the error probability to an arbitrarily small value. A user message is composed of *k* bits of information and transmitted through a noisy channel in one of $M=2^k$ messages. Each message $m=1,...,M$ is encoded through a function $f:\{1,2,..., M\} \mapsto \mathbb {R}^{n}$ to a sequence $\textbf{X}$ of size *n* denominated codeword, where *n* is referred to as blocklength. We denote the resulting code as an (*n*, *M*)-code. Also, we define $R=k/n$ as the transmission rate, with $k<n$. Once the codeword is transmitted over the noisy channel, the received vector is given by:

1 $$\begin{aligned} \textbf{Y} = \textbf{X}+\textbf{N}, \end{aligned}$$

where $\textbf{N} \sim \mathcal {N}(0,\textbf{I}_{n})$ is multidimensional Gaussian noise, which is assumed to have i.i.d. individual components with zero mean and $\textbf{I}_n$ denotes the $n \times n$ identity matrix. Also, to take into account the limited device battery life and regulatory constraints, we assume that the codewords are subject to the equal power constraint:

2 $$\begin{aligned} \left\| {{\textbf {X}}} \right\| _2^2 = n\rho , \end{aligned}$$

where $\rho =\textrm{PT}$, *P* is the received power and *T* is the duration of an entire codeword. Similar to the channel coding process, at the receiver, a decoding function $g: \mathbb {R}^{n} \mapsto \{1,2,..., M\}$ maps the received sequence $\textbf{Y}$ to one of the possible transmitted messages ${\hat{m}} \in \{1,...,M\}$ or else declares an error. If the additive noise has a unit variance, the signal-to-noise ratio (SNR) is equal to $\rho$. A codebook and a decoder whose SNR is equal to $\rho$ is called an $(n,M,\rho )$-code. Under these conditions and considering an infinite blocklength, the Shannon capacity in bits per channel uses for a discrete-time additive white Gaussian noise (AWGN) channel is given by:

3 $$\begin{aligned} C = \frac{1}{2} \log _{2}\left( 1+\rho \right) . \end{aligned}$$

The noisy channel coding theorem asserts that, for reliable communications, the transmission rate *R* cannot be greater than the capacity *C* [20]. Since the duration of an entire codeword is *T*, the latency can be expressed as $L=nT$. Hence, based upon the noisy channel coding theorem, for a given power $\rho$ and bandwidth *W*, the minimal latency is then given by:

4 $$\begin{aligned} {L_{\min }} =\frac{{{\log }_2}(M)}{W{{\log }_2}(1 + \rho )}. \end{aligned}$$

As indicated by (4), in and infinite-blocklength regime, the minimal latency $L_{\min }$ is reduced by increasing the bandwidth $W={1}/{2T}$ or the power $P={\rho }/{T}$.

#### Minimal achievable latency in the finite-blocklength regime

In specific modern applications (e.g., tactile internet), it is mandatory to use short packets to achieve the required latency and reliability constraints. In those cases, the error probability will no longer be arbitrarily low. Thus, a framework that relates packet error probability, communication rate, short packet size, and latency is essential. Toward this end, Polyanskiy et al. [8] established performance metrics for which the maximal code size achievable for the desired error rate and a fixed short blocklength can be tightly approximated for various channel types and conditions. Such a framework is called the finite blocklength (FBL) regime. Thus, with a finite blocklength, it is necessary to define the coding scheme by taking into account the average BLER constraint given by:

5 $$\begin{aligned} \Pr ({\hat{m}} \ne m) \le \epsilon , \end{aligned}$$

where the measure $\Pr$ denotes the conditional probability that the decoder ${\hat{m}}=g(\textbf{Y})$ has made an incorrect decision on the message, when the actual message *m* was transmitted. Thus, an $(n,M,\rho )$-code whose average BLER is not larger than $\epsilon$, as shown in (5), is denoted as an ($n,M,\rho ,\epsilon$)-code. For the FBL regime, the following theorem provides an upper bound of the maximal code size achievable for a given error probability $\epsilon$, finite blocklength *n*, and power constraint $\rho$ [8, Th. 54].

##### Theorem 3.1

(Polyanskiy et al.) For the AWGN channel with SNR $\rho$, capacity *C*, $0<\epsilon < 1$, and for equal-power and maximal-power constraints, the maximal code size achievable is given by:

6 $$\begin{aligned} \log _{2} M^{*}(n,\epsilon ,\rho ) = nC-\sqrt{n V(\rho )} Q^{-1}(\epsilon ) + O(\log _{2} n), \end{aligned}$$

where the error term is described by $O(\log _{2} n)$, $Q^{-1}(\cdot )$ denotes the inverse of the Gaussian *Q* function, and $V(\rho )$ is the channel dispersion^2^:

7 $$\begin{aligned} V(\rho ) = \frac{\rho }{2} \frac{\rho +2}{(\rho +1)^{2}} \log _{2}^{2}e, \end{aligned}$$

where *e* is Euler’s constant. In [8, 23], it has been shown that a good approximation for (6) is obtained if we replace the term $O(\log _{2} n)$ with ${1}/{2}\log _{2} n + O(1)$. Such approximation, referred to as normal approximation, is given by:

8 $$\begin{aligned} \log _{2} M^{*}(n,\epsilon ,\rho ) \approx nC-\sqrt{n V(\rho )} Q^{-1}(\epsilon ) + {\frac{1}{2}}{\log _2}n + O(1). \end{aligned}$$

Based on the bound of $O(\log _{2} n)$ in [8, Th. 54], we can determine the converse bound for equal-power and maximal-power constraints by:

9 $$\begin{aligned} \begin{aligned} \log _{2} M^{*}(n,\epsilon ,\rho ) \le nC-\sqrt{nV(\rho )} Q^{-1}(\epsilon ) + \frac{1}{2}\log _{2} n + O(1). \end{aligned} \end{aligned}$$

##### Remark 3.2

An explicit result in terms of physical variables that are linked to the channel can be obtained by introducing the latency *L*, the power *P*, and the symbol duration *T* in (9). Moreover, by expressing $L = nT$ and for a given bandwidth *W* (the Nyquist criterion states that $T = {1}/{2W}$), the converse bound is rewritten as:

10 $$\begin{aligned} \begin{aligned} \log _{2} M^{*}(n,\epsilon )\le&{\frac{L}{2T}}{\log _e}(1 + PT){ \frac{1}{{\log _e}2}}-\sqrt{\frac{L}{T} \frac{PT(PT+2)}{2(PT+1)^{2}}}\\&\cdot \log _{2}(e)Q^{-1}(\epsilon )+ \frac{1}{2}\log _{2}\left( \frac{L}{T}\right) +O(1). \end{aligned} \end{aligned}$$

##### Remark 3.3

By approximating the logarithmic functions, two observations are possible: when *PT* is small, (10) may be rewritten for a power-limited region:

11 $$\begin{gathered} \log _{2} M^{*} (n,\epsilon )\sim {\mkern 1mu} \frac{{LP}}{{2\log _{e} 2}} - \sqrt {LP} \log _{2} (e)Q^{{ - 1}} (\epsilon ) \hfill \\ + \frac{1}{2}\log _{2} \left( {\frac{L}{T}} \right) + O(1),{\mkern 1mu} {\mkern 1mu} {\mkern 1mu} {\text{as}}\;PT \to 0. \hfill \\ \end{gathered}$$

Under such a condition, the first two terms of the right side of (11) are independent of $T={1}/{2W}$ and the third term, rewritten as ${1}/{2} \log _2(2WL)$, shows that the maximal code size achievable tends to be constant as *W* grows. Thus, in a power-limited region, increasing the bandwidth does not significantly augment the maximum achievable code size, especially when $PT\ll 1$. On the other hand, when $PT\gtrsim 2$, Eq. (10) may be rewritten for a bandwidth-limited case:

12 $$\begin{gathered} \log _{2} M^{*} (n,\epsilon )\sim {\mkern 1mu} \frac{L}{{2T}}\log _{2} (1 + PT) - \sqrt {\frac{L}{{2T}}} \log _{2} (e)Q^{{ - 1}} (\epsilon ) \hfill \\ + \frac{1}{2}\log _{2} \left( {\frac{L}{T}} \right) + O(1),{\mkern 1mu} {\mkern 1mu} {\mkern 1mu} {\text{as}}\;PT{ \gtrsim }2. \hfill \\ \end{gathered}$$

As for the classical capacity Eq. (3), (12) shows that the reduction in latency is linked to the bandwidth and power under normal approximation.

### Early detection using sequential tests

From here on, we define communication latency as the delay between the beginning of the transmission of a message and the instant at which the receiver makes a correct decision. Ignoring the propagation delay, the latency can be smaller than the symbol duration if an early detection mechanism is used [19].

#### Problem formulation

We consider a transmitter that sends a message with a fixed symbol duration *T*. We take equidistant samples of this symbol and form a collection of i.i.d. random vectors $\textbf{Y}_T = \left\{ \textbf{Y}_{1},\textbf{Y}_{2}, \cdots , \textbf{Y}_{\lambda }\right\}$ with a distribution *f*, where $\lambda$ is an integer equal to the total number of samples. That is, each sample is taken at a distance $d = T / \lambda$. A sequential test uses a subset $\kappa$ of these $\lambda$ samples to determine the transmitted message. Such a subset of samples is denoted as ${{{\textbf {Y}}}_t} = \left\{ {{{{\textbf {Y}}}_1},{{{\textbf {Y}}}_2},...,{{{\textbf {Y}}}_\kappa }} \right\}$, where $\kappa \le \lambda$. Considering a channel model with additive noise, each $\textbf{Y}_{i}$ is given by:

13 $$\begin{aligned} \textbf{Y}_{i} = \textbf{X}_{i}+\textbf{N}_{i}\,, \end{aligned}$$

where $\textbf{X}_{i} \in \mathbb {R}^{n}$ is the message of blocklength *n* indexed by $i=1,2,...,\lambda$ and $\textbf{N}_{i} \sim \mathcal {N}(0,\textbf{I}_{n})$ is the AWGN vector. Such a message signal satisfies the equal-power constraint denoted by:

14 $$\begin{aligned} \left\| {{{{\textbf {X}}}_i}} \right\| _2^2 = n{\rho _i}\,, \end{aligned}$$

where $\rho _{i} = P\tau _{i}$, and $\tau _{i}$ is a small proportion of the symbol duration *T*. Specifically, when $\rho = \textrm{PT}$, we have:

15 $$\begin{aligned} \sum \limits _{i = 1}^\lambda {\left\| {{{{\textbf {X}}}_i}} \right\| _2^2 = n\rho }\,. \end{aligned}$$

In this paper, we assume that the received signal has been projected onto the orthogonal basis to obtain $\textbf{Y}_{i}$. This means that the bases spanning the vector-space of signals are orthonormal for all the proportions of the symbol duration $\tau _{i}$.

If we decide which message was sent using only a small proportion of the duration of the transmitted symbol, we can reduce latency. To do so, we leverage on one of the quick decision techniques called sequential test [9–11]. Such a sequential test decides which message was transmitted based on a likelihood ratio that depends on the samples and distribution of the message. For the sake of simplicity, we assume that the *M* transmitted messages are equiprobable, i.e., the a priori distribution is constant [20, 24]. Thus, the optimal decision on which message was sent with respect to a finite $\kappa$ number of samples is performed through a sequential probability ratio test, which is formulated as follows:

16 $$\begin{aligned} g\left( {{{{\textbf {Y}}}_t}} \right) = m,\;\mathrm{{if}}\;\exists m\;\mathrm{{s}}\mathrm{{.t}}.\;\Pr [m|{{{\textbf {Y}}}_t}] > {S_m}. \end{aligned}$$

In other words, we stop as soon as the posterior probability $\Pr [m|{{{\textbf {Y}}}_t}]$ exceeds a threshold $S_{m}$ for some *m*, and decide that *m* was transmitted. Otherwise, data reception continues. By using the proposed EDS, latency is reduced when the message is received without errors at a time $\tau$ shorter than the symbol duration *T*. It should be noted that if the posterior probability maximized for some *m* does not exceed the threshold $S_{m}$, the decision is made at *T*. In Sect. 3.2, we show that the threshold $S_m$ is a function of both the conditional probability of error and the prior probability of the transmitted message.

#### Minimal achievable latency using an optimal early-detection scheme

An EDS is optimal if it minimizes the expectation of the proportion of the symbol duration used for detection for which the error rate does not exceed a predefined value. For such a scheme, we assume that a perfect error-detecting code checks whether or not there are errors in the transmitted message, i.e., the undetected error probability is zero. In that case, the decision rule is as follows: as soon as an error has not been detected, decode the message. Otherwise, wait for the next sample until no error occurs. If errors are detected up until the end of the transmitted symbol *T*, the decision will be made at *T*.

Under such a condition, the average latency can be determined by the error rate as a function of the SNR $\rho$. To this end, we use an arbitrary ($n,M,\epsilon ,\rho$)-code whose average BLER is not larger than $\epsilon$, and subject to a power constraint $\rho$. By using Theorem 3.1, we obtain the maximum achievable channel code rate $R^{*}(n,\epsilon ,\rho )$ (in bits per channel use) such that:

17 $$\begin{aligned} R^{*}(n,\epsilon ,\rho ) = C-\sqrt{\frac{V(\rho )}{n}}Q^{-1}(\epsilon )+\frac{1}{2n} \log _{2} n + O(1)\,. \end{aligned}$$

It follows that for a given fixed channel code rate *R*, SNR $\rho$, and blocklength *n*, the non-asymptotic error-correction performance of such codes can be determined solving (17) for $\epsilon$:

18 $$\begin{aligned} \begin{aligned} \epsilon ^{*}(\rho ,R,n)&= Q\left( \frac{C-R+ \frac{1}{2n}\log _{2} n + O(1)}{\sqrt{V(\rho )/n}}\right) \\&\approx Q\left( \frac{C-R+ \frac{1}{2n}\log _{2} n}{\sqrt{V(\rho )/n}}\right) \,. \end{aligned} \end{aligned}$$

The approximation provided by (18) is accurate for $n> 100$ since the *O*(1) term is a small constant, and the term $\frac{1}{2n}\log _{2} n$ quickly becomes negligible as *n* is greater than 100 channel uses [8, 25, 26].

##### Remark 3.4

With *C* being expressed as (3), the minimum proportion of symbol duration required to reliably obtain a message is provided by Shannon’s noisy channel coding theorem $R < C$, such that:

19 $$\begin{aligned} { \frac{{2^{2R}} - 1}{P} }< \tau < T\,, \end{aligned}$$

where $\tau$ is the early detection time in which the scheme performs a reliable detection.

For the FBL regime, the following theorem provides the optimal average latency of the EDS. For clarity, we denote an $(n,M, \epsilon )$-code, whose average latency is equal to $\bar{\tau }$, as an (*n*,*M*,$\epsilon$,$\bar{\tau }$)-code.

##### Theorem 3.5

The optimal average latency of the EDS for an arbitrary (*n*,*M*,$\epsilon$,$\bar{\tau }$) code is given by:

20 $$\begin{aligned} {\bar{\tau }} \le { \frac{1}{\sqrt{2\pi }}}\int _0^T {\tau \exp \left[ {{\frac{ - {\gamma }^2 {(\tau )}}{2} }} \right] d\gamma (\tau )}\,, \end{aligned}$$

with $\gamma (\tau )$ defined as:

21 $$\begin{aligned} \gamma (\tau ) = \frac{C(P\tau )-R+ \frac{1}{2n}\log _{2} n}{\sqrt{V(P\tau )/n}}\,, \end{aligned}$$

where the capacity $C(P\tau )$ and the channel dispersion $V(P\tau )$ are two time-dependent functions given that $\rho$ in Eqs. (3) and (7) is replaced by $P\tau$. Note that $\gamma (\tau )$ results from isolating $\epsilon ^{*}$ in (17) as shown in the argument of (18).

This theorem can be proved by using the following lemma.

##### Lemma 3.6

For an AWGN channel with an arbitrary ($n,M,\epsilon ,\bar{\tau }$)-code whose error probabilities satisfy (18) and for all $d \tau > 0$, the distribution of $\tau$ for an optimal EDS is given by the differential of the error [13]:

22 $$\begin{aligned} \begin{aligned} \lim \limits _{d \tau \rightarrow 0} p(\tau +d \tau )&= -\frac{d Q(\gamma (\tau ))}{d\gamma (\tau )}d\gamma (\tau )\\&= \frac{1}{\sqrt{2\pi }}e^{-\gamma ^{2}(\tau )/2}d\gamma (\tau )\,. \end{aligned} \end{aligned}$$

##### Proof

**Fig. 1:**
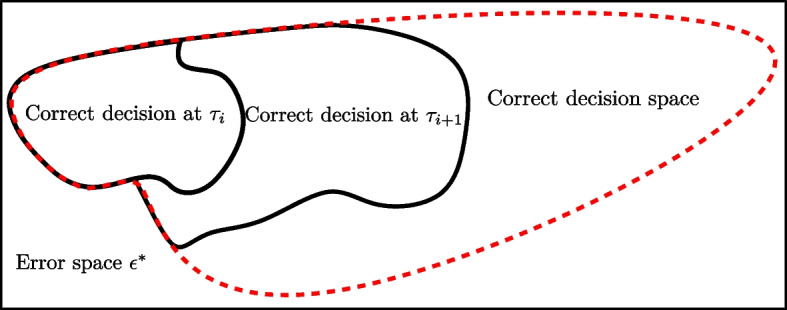
Decision in an optimal early-detection scheme using a perfect error-detection code, where the correct detection at $\tau _{i}$ or $\tau _{i+1}$ are mutually exclusive events

Let us consider that the receiver performs an early detection at $\tau _{1}$ and $\tau _{2}$ subject to $\tau _{1} < \tau _{2} \le T$ where a perfect error-detecting code checks whether or not there are errors in the message. For the sake of simplicity, we define $\Pr [g(\textbf{Y}_{\tau _1})=m|M=m,\tau _1]$, i.e., the probability of having a correct decision at $\tau _1$, as $p(\tau _1)$. Thus, $p(\tau _{1}) = 1-\epsilon ^{*}(P\tau _{1},R,n)$. If the decoder has not decided at $\tau _{1}$ it means that there is an error in the message and we wait for the next sample at $\tau _{2}$. Therefore, the probability to make a correct decision at $\tau _{2}$ depends on the probability of having a correct decision previously, i.e, $p({\tau _2}) = \Pr [g({{{\textbf {Y}}}_{{\tau _2}}}) = m|M = m,{\tau _2},g({{{\textbf {Y}}}_{{\tau _1}}}) \ne m]$. Indeed, if the decoder has decided at $\tau _{2}$, then it means that errors have been detected previously. Thus, since $p(\tau _1)$ and $p(\tau _2)$ are mutually exclusive events, the probability of having a correct decision at $\tau _{2}$ is $p(\tau _{2}) = (1-\epsilon ^{*}(P\tau _{2},R,n))-(1-\epsilon ^{*}(P\tau _{1},R,n)) = \epsilon ^{*}(P\tau _{1},R,n)-\epsilon ^{*}(P\tau _{2},R,n)$. Figure 1 illustrates the decision rule in an optimal EDS using a perfect error-detecting code.

Hence, we could thus generalize our purpose by letting $S_{\tau } = \left\{ \tau _{1}, \tau _{2},\cdots , T\right\}$ be an increasing and positive sequence of samples that are used to make a decision on the message. In addition, we consider that there exists a $\tau + d \tau \in S_{\tau }$ such that the probability of having a correct decision at $\tau + d\tau$ is given by:

23 $$\begin{aligned} \begin{aligned} p(\tau +d\tau )&= (1-\epsilon ^{*}(P(\tau +d\tau ),R,n))-(1-\epsilon ^{*}(P\tau ,R,n))\\&= \epsilon ^{*}(P\tau ,R,n)-\epsilon ^{*}(P(\tau +d\tau ),R,n)\,. \end{aligned} \end{aligned}$$

If we rewrite (23) in the form:

24 $$\begin{aligned} p(\tau + d\tau ) = -{\frac{({\epsilon ^*}(P(\tau + d\tau ),R,n) - {\epsilon ^*}(P\tau ,R,n))}{d\tau }}d\tau \,, \end{aligned}$$

where the fraction on the right side is the average slope, Eq. (24) states that the probability of a correct decision in an interval near $\tau$ is the average slope over the interval times the length of that interval. By definition, the limit of the average slope is the derivative of the error $\epsilon ^*$ evaluated at $\tau$ as $d\tau \rightarrow 0$.

Hence, since any (*n*,*M*,$\epsilon$,$\bar{\tau }$) code satisfies (18), the distribution of $\tau$ can be simplified as in (22) by using Eqs. (18) and (21) in (24), and by letting $d\tau \rightarrow 0$. The average latency of an ($n,M,\epsilon ,\bar{\tau }$) code is given by the expectation of $\tau$ as in (20). This concludes the proof. $\square$

In the following section, we design the EDS based upon a sequential test. The test employs a list decoder in which the MSPRT can make a decision based on a few hypotheses.

#### On the design of early-detection scheme: an MSPRT scheme guided by list decoding

Consider $M=2^{k}$ possible messages of *k* bits encoded by an arbitrary ($n,M,\epsilon$)-code whose each symbol has a fixed duration *T*. Also consider that all symbols of the encoded message $m \in \left\{ {1,2,...,M} \right\}$ are simultaneously transmitted through a parallel AWGN channel with *n* branches. The MSPRT allows for latency reduction by choosing which message was transmitted among *M* possible messages as soon as the probability of its correct detection is high enough to exceed a given threshold $S_m$. Inspired by previous works on sequential detection [9, 10, 18, 27, 28], the early-detection problem can be formulated for multidimensional signaling. By using Bayes’ rule, the posterior probabilities in (16) can be written as:

25 $$\begin{aligned} \Pr [M = m|{{{\textbf {Y}}}_1},{{{\textbf {Y}}}_2},...,{{{\textbf {Y}}}_\kappa }] = \frac{\pi _{m} \prod \limits _{i=1}^{\kappa } f(\textbf{Y}_{i}|m)}{\sum \limits _{j = 1}^{M}\pi _{j} \prod \limits _{i=1}^{\kappa } f(\textbf{Y}_{i}|j)}\,, \end{aligned}$$

where $\pi _{j}$ is the prior probability of the transmitted message, $f(\textbf{Y}_{i}|j)$ is the likelihood function for $j = 1,2\cdots , M$. Hence, the stopping time $\tau _{m}$ and the decision rule $\delta$ is given by:

26a $$\begin{aligned} {\tau _m}&= \inf \left\{ {t=\kappa d:\; \text {if}\; \exists \; m\; \text {s.t.} \; \Pr [M = m|{{{\textbf {Y}}}_1},{{{\textbf {Y}}}_2},...,{{{\textbf {Y}}}_\kappa }] > {S_m}} \right\} \,, \end{aligned}$$

26b $$\begin{aligned} \delta&= {\hat{m}},\;\mathrm{{where}}\;{\hat{m}} = \arg \;\mathop {\max }\limits _{1 \le m \le M} \left( {{\pi _m}\prod \limits _{i = 1}^\kappa {f\left( \textbf{Y}_{i}|m \right) } } \right) \,, \end{aligned}$$

where $0 \le S_{m} \le 1$. Such equations mean that the receiver stops the reception of the samples as soon as the posterior probability exceeds a threshold and decides which *m* was transmitted. It should be noted that if the posterior probability maximized for some *m* does not exceed the threshold $S_{m}$, the decision is made at *T*.

The performance of the system is given by the average of the message error probabilities:

27 $$\begin{aligned} \epsilon = \sum \limits _{m = 1}^{M}\pi _{m}\text {P}_{\textbf{Y}\mid m}[g(\textbf{Y}_{\tau })\ne m |M = m]\,, \end{aligned}$$

where $\text {P}_{\textbf{Y}\mid m}[g(\textbf{Y}_{\tau }) \ne m]$ is the probability of error when the sequential test stopped at $\tau < T$ and chose the wrong message. In [9, 10], it has been proven that (27) has an upper bound for a given threshold $S_{m}$. This is given by the following theorem.

##### Theorem 3.7

(Baum and Veeravelli) Let $\epsilon _{m',m} = \text {P}_{\textbf{Y}\mid m'}[g(\textbf{Y}_{\tau })= m]$ be the probability of deciding that message *m* was transmitted when $m'$ was actually sent, and $\epsilon _{m} = \text {P}_{\textbf{Y}\mid m}[g(\textbf{Y}_{\tau })\ne m]$ the probability of incorrectly deciding that message *m* was transmitted. Then $\epsilon _{m',m}$ and $\epsilon _{m}$ are upper bounded:

28a $$\begin{aligned} \epsilon _{m}&= \sum \limits _{\begin{array}{c} m'=1 \\ m'\ne m \end{array}}^{M} \pi _{m'}\epsilon _{m',m} \le \pi _{m} \frac{1-S_{m}}{S_{m}}\,, \end{aligned}$$

28b $$\begin{aligned} \epsilon&= \sum \limits _{m = 1}^{M} \epsilon _{m} \le \sum \limits _{m = 1}^{M}\pi _{m} \frac{1-S_{m}}{S_{m}}\,, \end{aligned}$$

28c $$\begin{aligned} \epsilon&\le {{1 - S}\over S} ~\text {if}~ S = S_{1} = S_{2} = \cdots = S_{M}\,. \end{aligned}$$

From Theorem 3.7, it follows that the threshold $S_{m}$ can be written as:

29 $$\begin{aligned} S_{m} \le \frac{1}{1+ {\pi }_{m}^{-1}\sum \limits _{\begin{array}{c} m'=1 \\ m'\ne m \end{array}}^{M} {\pi }_{m'}\epsilon _{m',m}}\,. \end{aligned}$$

It can be noted from (29) that the threshold $S_{m}$ is chosen to meet the error probability constraint $\epsilon$. Therefore, $S_{m}$ must be defined such that the latency is minimized while ensuring that $\text {P}_{\textbf{Y}\mid m}[g(\textbf{Y}_{\tau })\ne m]$ does not exceed a predefined value.

In the next section, we show through examples that the EDS can be designed via sequential tests based on list-decoding combined with MSPRT.

### Examples of the early-detection scheme for low-latency communication

Examples of the EDS for low-latency communication are provided in this section. Noteworthy results on both latency and error probabilities are discussed in Sect. 4 under various channel conditions and blocklengths.

#### Low-latency communication under additive white Gaussian noise channels

Considering a message of *n* symbols that are transmitted simultaneously through a parallel AWGN channel with *n* branches. There are $M = 2^{k}$ uniformly distributed possible messages. Each symbol is transmitted with a fixed duration *T* and is modulated by a binary phase-shift keying (BPSK). The signal messages are denoted by $\textbf{X}^{m} \in \mathbb {R}^{n}$ for all $m = 1,2,\cdots , M$ which satisfy (2). The receiver observes a small proportion of the signal message denoted by a sequence ${{{\textbf {Y}}}_t} = \left\{ {{{{\textbf {Y}}}_1},{{{\textbf {Y}}}_2},...,{{{\textbf {Y}}}_\kappa }} \right\}$ of independent Gaussian variables with mean $\textbf{X}_{i}^{m}$ where $i = 1,2, \cdots , \kappa$ whose satisfies (14). The variance of the noise is constant and denoted by $\frac{N_{0}}{2}\textbf{I}_{n}$.

For a large information block size, the receiver needs $2^{k}$ tests in order to choose which message has the largest posterior probabilities. Thus, such a sequential test might be challenging to implement for very large information block sizes [12]. Interestingly, a list decoder can significantly reduce the number of hypotheses for sequential tests by providing a list of the $\ell < M$ most probable messages. Since $\ell$ is less than *M*, prior probabilities for $\ell$ most probable possible messages should be redefined as $\bar{\pi }_{m} = \pi _{m}/(\sum _{j = 1}^{\ell } \pi _{j})$, which renders (26) accurate. For early detection using MSPRT under an AWGN channel, such a test takes the form of (30).

30a $$\begin{aligned} \tau _{m}&= \inf \left\{ t: \sum \limits _{\begin{array}{c} m'=1 \\ m'\ne m \end{array}}^{\ell } \exp \left( \sum \limits _{i = 1}^{\kappa } \frac{\left( \textbf{X}^{m'}-\textbf{X}^{m}\right) ^{\text {tr}}\textbf{Y}_{i}}{\frac{N_{0}}{2}} \right) < \frac{1-S_{m}}{S_{m}} \right\} \,, \end{aligned}$$

30b $$\begin{aligned} \delta&= {\hat{m}},\;\mathrm{{where}}\;{\hat{m}} = \arg \mathop {\min }\limits _{1 \le m \le M} \left\| {{{{\textbf {Y}}}_{{\tau _m}}} - {{{\textbf {X}}}^m}} \right\| \,, \end{aligned}$$

where $(\cdot )^{\text {tr}}$ denotes the transpose of a vector.

Since the threshold $S_{m}$ is linked to the error probability constraint $\epsilon$, such an optimal threshold may be difficult to obtain due to the modulation scheme and channel conditions. However, we have conjectured a simple method to establish such a threshold for the equiprobable binary signaling system considered for the EDS. The advantage of this threshold is that it requires only the evaluation of pairwise error probabilities. Inspired by previous results on MSPRT in [9, 10, 15, 29], the threshold $S_{m}$ in (29) can be defined as:

31 $$\begin{aligned} S_{m} = \frac{1}{1+\ell \sum \limits _{\begin{array}{c} m = 1 \\ m\ne m' \end{array}}^{\ell }P_{e}(m\rightarrow m')}\,, \end{aligned}$$

where $P_{e}(m\rightarrow m')$ is the pairwise error probability, the probability that the receiver chooses $\textbf{X}^{m'}$ over $\textbf{X}^{m}$ when $\textbf{X}^{m}$ was transmitted. For $\ell = 2$, the test becomes Wald’s SPRT because there are two possible messages.

##### Remark 3.8

For AWGN channels, and under normal distribution, $P_{e}(m\rightarrow m')$ is given by:

32 $$\begin{aligned} P_{e}(m\rightarrow m') = Q\left( \frac{\Vert \textbf{X}^{m}-\textbf{X}^{m'} \Vert }{\sqrt{2 N_{0}}}\right) \,, \end{aligned}$$

where $N_{0}/2$ is the variance of the noise [20]. However, it will be necessary for other modulation schemes and channel models to use the union bound (which is the simplest and most widely used bound) to compute the error probability used on the threshold selection. Such a bound is quite tight, especially at high signal-to-noise ratios.

#### Early detection scheme with OFDM

As shown above, early detection via a sequential tests reduce latency if symbols are transmitted in parallel. A typical parallel transmission is OFDM signaling because data is transmitted in parallel by mapping each symbol to a carrier [20]. OFDM is efficient only if the subcarrier spacing is proportional to the inverse of symbol duration *T*, which guarantees orthogonality [13]. A critical question that needs to be explored: can we decide faster which message was sent by evaluating distances between different OFDM signals? To answer this, consider a codeword $\textbf{X}^{m} \in \mathbb {C}^{n}$ arbitrarily chosen among *M* possible codewords, and its OFDM signal $s_{m}(t)$:

33 $$\begin{aligned} s_{m}(t) = \sum _{k = 1}^{n} X_{k}^{m}e^{\frac{j2\pi kt}{T}}, \end{aligned}$$

where $X^{m}_{k}$ is the information signal. For OFDM, an EDS can be defined as follows: the receiver quickly make a reliable decision as soon as the distance between the received noisy signal *y*(*t*) and $s_{m}(t)$ reaches a threshold $S_{m}$. Hence, the stopping rule can be defined as:

34a $$\begin{aligned} \tau _{m}&= \inf \left\{ t: \; \text {if}\; \exists \; m\; \text {s.t.} \; \Vert y(t)- s_{m}(t) \Vert < S_{m} \right\} \,, \end{aligned}$$

34b $$\begin{aligned} \delta&= \hat{m},~ \text {where}~ \hat{m} = {\mathop {\textrm{arg}\,\textrm{min}}\limits _{1\le m\le M}}\Vert y(\tau _{m})- s_{m}(\tau _{m}) \Vert \,. \end{aligned}$$

There are several noteworthy features on the distances of these OFDM signals.

##### Remark 3.9

Assuming a random coding where $\textbf{X}^{m}$ and $\textbf{X}^{m'}$ are i.i.d. random vectors, the squared distance between $s_{m}(t)$ and $s_{m'}(t)$ is given by:

35 $$\begin{aligned} \begin{aligned} \left( d_{t}^{mm'}\right) ^{2}&= \Vert \textbf{X}^{m} \Vert ^{2}t+\Vert \textbf{X}^{m'} \Vert ^{2}t-2\Re \left( \int _{0}^{t} s_{m}(\xi ) s_{m'}^{*}(\xi ) d\xi \right) \,, \end{aligned} \end{aligned}$$

where $\Re \left( \cdot \right)$ denotes the real part of a complex number. Since $\textbf{X}^{m}$ and $\textbf{X}^{m'}$ are independent, then the covariance is null. Thus, the squared distance is approximately linear over time. As a result, it is possible to use early detection efficiently when random coding schemes are employed.

However, codewords are generally non-i.i.d. random vectors. Consider a codeword $\textbf{X}^{m}$ and its nearest neighbor $\textbf{X}^{m'}$ such that the squared distance over time is given by:

36 $$\begin{aligned} \begin{aligned} \left( d_{t}^{mm'}\right) ^{2}&= \int _{0}^{t} \bigg \vert \sum \limits _{k \in \mathcal {K}}\left( X_{k}^{m}-X_{k}^{m'}\right) e^{j2\pi \frac{k}{T}\xi }\bigg \vert ^{2} d\xi \,, \end{aligned} \end{aligned}$$

where $\mathcal {K}$ is a subset in which $X_{k}^{m}-X_{k}^{m'} \ne 0, \forall k \in \mathcal {K}$, otherwise $\left( d_{t}^{mm'}\right) ^{2}$ is equal to zero. As a result, the distances between OFDM signals are nonlinear functions over time which could render the EDS not efficient. This non linearity is due to dimensions that overlap each other $\forall t \in [0,T)$. For example, consider a codebook of *M* codewords that are mapped to quadrature phase-shift keying (QPSK) modulation, in which there are at most two different symbols among these *n* dimensions, i.e., the number of elements in $\mathcal {K}$ is equal to one or two. In such a case, we observe these following remarks.

##### Remark 3.10

We may use fewer subcarriers to increase the space between subcarriers; hence, the system remains orthogonal. However, for the band-limited OFDM system, the orthogonality holds for specific samples. For instance, with a subcarrier spacing of 4/*T*, the system is orthogonal at 0.25*T*, 0.5*T*, 0.75*T* and *T*. In other words, such an increase in subcarrier spacing would be convenient if the early detection could be guaranteed at $50\%$ or $75\%$ of the symbol duration.

##### Remark 3.11

Latency can be reduced through EDS if the squared distance $\left( d_{t}^{mm'}\right) ^{2}$ is linear for all $m \ne m'$ in an arbitrary codebook. To do so, it is possible to use pre-coding random rotation matrices to linearize these distances. Considering a complex matrix $\textbf{H}$ whose angles are i.i.d., and viewing $A_{l}^{mm'} = X_{l}^{m}-X_{l}^{m'}$, Eq. (36) can be rewritten as:

37 $$\begin{aligned} \begin{aligned} \left( d_{t}^{mm'}\right) ^{2}&= \int _{0}^{t} \bigg \vert \sum \limits _{k \in \mathcal {K}} \sum \limits _{l}H_{k,l}A_{l}^{mm'}e^{j2\pi \frac{k}{T}\xi }\bigg \vert ^{2} d\xi \,, \end{aligned} \end{aligned}$$

where $H_{k,l}$ is an element of $\textbf{H}$. Denoting $\mathcal {K} = \left\{ k_{1},k_{2} \right\}$, we obtain (38) where $H_{k_{2},l}^{*}$ is the complex conjugate of the element $H_{k_{2},l}$. Since $\textbf{H}$ is a random rotation matrix in which elements are i.i.d., $\sum _{l} H_{k_{1},l} H_{k_{2},l}^{*}$ must be zero. Hence, the squared distance $\left( d_{t}^{mm'}\right) ^{2}$ becomes linear.

38 $$\begin{aligned} \begin{aligned} \left( d_{t}^{mm'}\right) ^{2}&= \left( \sum \limits _{l} \bigg \vert H_{k_{1},l}A_{l}^{mm'} \bigg \vert ^{2} + \sum \limits _{l}\bigg \vert H_{k_{2},l}A_{l}^{mm'} \bigg \vert ^{2}\right) t \\&+2\Re \left( \int _{0}^{t} \sum \limits _{l} H_{k_{1},l} H_{k_{2},l}^{*}\bigg \vert A_{l}^{mm'} \bigg \vert ^{2} e^{j2\pi \frac{k_{1}-k_{2}}{T}\xi }d\xi \right) . \end{aligned} \end{aligned}$$

It should be noted that there are orthogonal matrices that meet such a condition. A Hadamard matrix is a typical example in which $\left( d_{t}^{mm'}\right) ^{2}$ can be linear.

#### Optimal latency for low-traffic multi-hop systems

In this example adapted from [13], the EDS is applied in low-traffic multi-hop systems, where short messages are re-transmitted as soon as the relay correctly decoded the message. For this purpose, we consider a decode-and-forward (DF) relaying scheme composing of *h* hops with synchronous detection (SD), i.e., detection at the end of the symbol duration. A transmitted codeword has latency $L=nT$ in each hop and latency is given by:

39 $$\begin{aligned} L_{\text {SD-DF}} = Lh\,. \end{aligned}$$

If relays make a reliable decision in a instant $\tau$ before the duration of the entire message *T*, the latency in (39) will be reduced. An EDS could make such a decision.

##### Theorem 3.12

In low-traffic multi-hop DF relaying system, assuming that the DF relays use an $(n,M,\epsilon )$-code along with an optimal EDS, if a relay transmits a message to the next relay through an OFDM-like signal, the minimal average latency using early-detection is upper bounded by:

40 $$\begin{aligned} \mathbb {E}\left[ L_{\text {ED-DF}}\right] \le L_{\text {SD-DF}}\,. \end{aligned}$$

##### Proof

To determine the latency for a multihop system using an optimal EDS, we consider *h* hops, where the $(n,M,\epsilon )$-code is transmitted by a source *S*, given a latency $L_{0} = nT$. The next a relay $R_{1}$ performs an optimal EDS, i.e., deciding at $\tau _{1} \le T$, given an accumulated delay equal to $L_{1} = L_{0}+\tau _{1}n$, and so forth. If each hop performs early detection at time instants $\left\{ {{\tau _1},{\tau _2},...,{\tau _h}} \right\} \le T$ and assuming that these time instants are an i.i.d. random sequence given that the Gaussian channel distribution is the same for every hop, the average latency is given by:

$$\begin{aligned} L_{\text {ED-DF}}&= {L_0} + {\tau _1}n + {\tau _2}n + ... + {\tau _{h - 1}}n\,,\\ \mathbb {E}\left[ L_{\text {ED-DF}} \right]&= nT + \mathbb {E}\left[ \sum \limits _{i = 1}^{h-1} \tau _{i}n \right] \\&= nT + (h-1)n\mathbb {E}\left[ \tau \right] \le hnT\\&\le L_{\text {SD-DF}}\,. \end{aligned}$$

The above demonstrates that the EDS can reduce latency in multi-hop systems using DF relaying schemes. $\square$

## Results and discussion

**Fig. 2:**
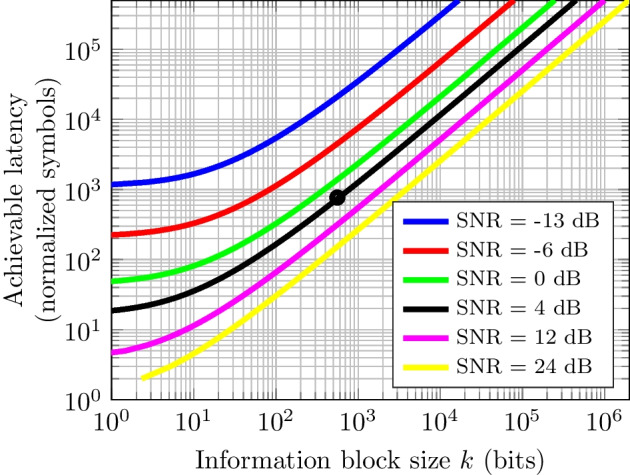
Achievable latency as a function of the information block size for different channel conditions. Probability of block error $\epsilon = 10^{-5}$. For example, at 4 dB, a message of $k=584$ bits has a latency of $L=7.67\,\mu$s (black round marker)

For a fixed-duration transmission, the minimal latency analyzed in Sect. 3.1.2 is obtained by (10). For a single-hop wireless link, and assuming a known constant channel gain,^3^ the achievable latency is depicted in Fig. 2 for a BLER of $\epsilon = 10^{-5}$. For example, let us consider that we have $PT = 4$ dB, and the bandwidth is $W = 50$ MHz. From Fig. 2, it can be seen that to transmit an information block size of $k=584$ bits, the achievable latency (in normalized symbols, $n=L/T$) is 767. Such a latency is conveniently expressed in time units as $L = 7.67~\mu$s, given that $L=n/2W$. For the parameter range considered here, the *O*(1) term in (10) is negligible compared to the other terms because *O*(1) is a small constant [21, 26].

Thus, following (10), it can be seen that the number of channel uses *n* decreases for a given *k* as the SNR increases. Such a trend is congruent with what we stated in Sect. 3.3.1, i.e., the latency for a system operating in the infinite-blocklength regime is reduced with increasing bandwidth, power, or both. Thus, Fig. 2 shows the achievable latency reduction when a system works in the short-blocklength regime.

**Fig. 3:**
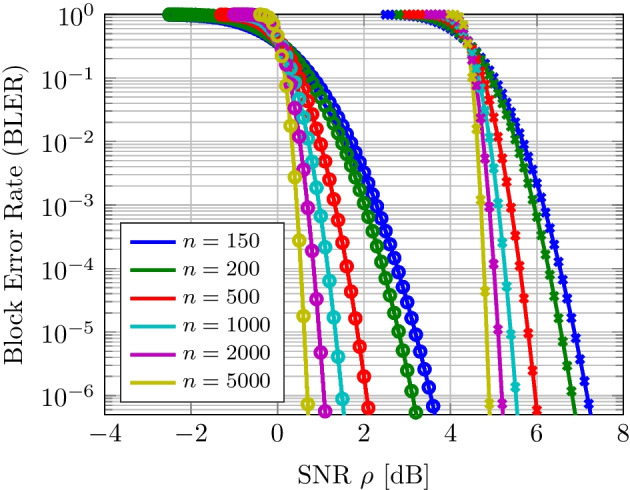
Non-asymptotic error performance of ($n,M,\epsilon ,\rho$) codes in the finite-blocklength regime. Round and cross markers correspond to code rates *R* of 0.5 and 0.95, respectively

An analysis of the codes whose latency can be reduced using a sequential test for a given error rate $\epsilon$ was provided in Sect. 3.2.2. Figure 3 shows the behavior of (18) of such ($n,M,\epsilon ,\rho$)-codes for different blocklengths *n* and code rates $R \in \{0.5, 0.95\}$ in bits per channel use. It can be seen that while all codes result in a BLER of 1 when the SNR is too low, increasing the blocklength quickly results in near-asymptotic error performance. For example, at $n=500$, for an SNR of approximately 2 dB and $R=0.5$, the error performance is already almost asymptotic. Following the normal approximation, the error performance improves as *n* grows since the term $\frac{1}{2n}\log _{2} n$ quickly vanishes for *n* greater than 100, resulting in asymptotic error behavior [8, 25, 26].

**Fig. 4:**
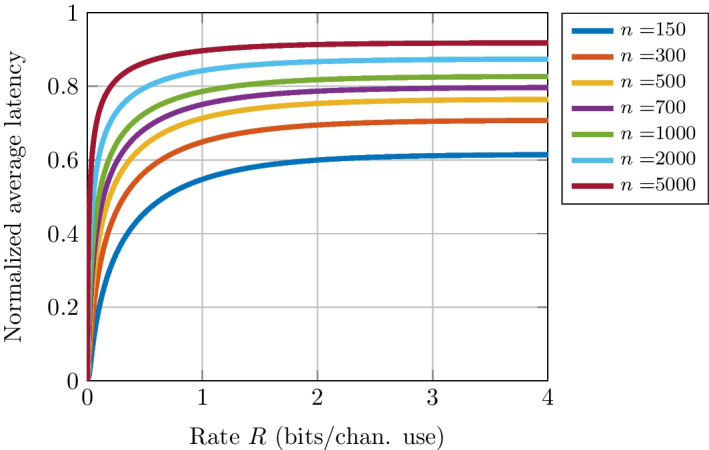
Normalized average latency as a function of channel code rate for various blocklength *n* and a block error rate (BLER) $\epsilon = 10^{-5}$

**Fig. 5:**
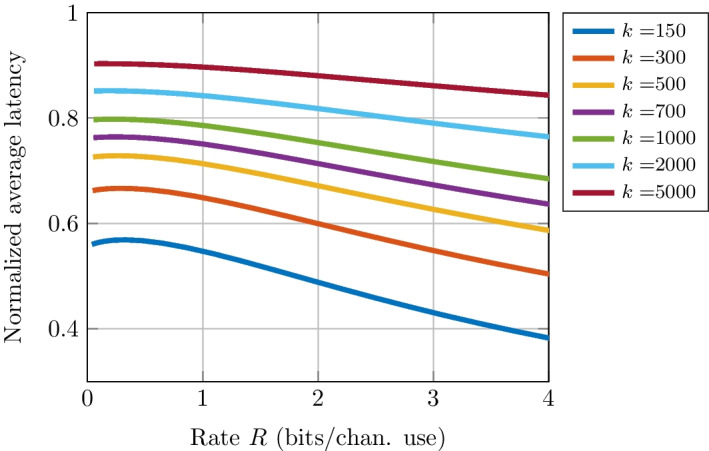
Normalized average latency as a function of channel code rate for various information block size *k* and a BLER $\epsilon = 10^{-5}$

**Fig. 6:**
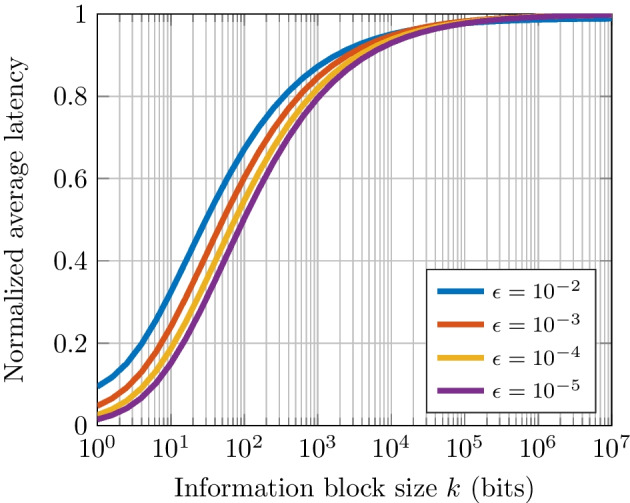
Normalized average latency as a function of the information block size *k* and rate $R = 0.5$ for various $\epsilon$

The normalized average latency of these codes is computed using Theorem 3.5 for various code sizes under varying channel conditions. Results are presented in Figs. 4, 5, 6. For a fixed blocklength *n*, Fig. 4 shows that as the rate increases, i.e., as the information-block size *k* grows, the symbol time required to receive messages becomes larger. Nevertheless, it is interesting to note that for a blocklength of $n=500$, messages can be sent with an error rate of $10^{-5}$ using $63\%$ and $71\%$ of the symbol time for rates *R* of 0.5 and 0.95 bit per channel use, respectively. Figure 4 also shows that an EDS allows for more significant latency reduction at shorter blocklengths. Indeed, for a fixed rate (say $R=0.5$), as the blocklength *n* grows, the required SNR to reach the necessary error performance decreases. Furthermore, when $R > C$, the error rate is close to 1 and the normalized average latency increases.

Figure 5 provides the normalized average latency for a fixed information block size *k*. On the one hand, it can be seen that the average latency decreases when the code rate increases. In other words, for a fixed information block size, a large blocklength increases latency slightly. On the other hand, for a fixed *R*, a large information block size increases latency. Indeed, it can be seen in this figure that for a rate of $R = 0.5$, $90\%$ of the symbol time on average is needed whereas $56\%$ of the symbol time is required for an information block size of $k = 5000$ and $k = 150$ bits, respectively. These results show that the minimal latency is not only linked to the blocklength, but mostly to the information block size.

The initial increasing behavior of the curves in Fig. 5 can be attributed to the fact that the formulas used are approximations, specifically the normal approximation (Eq. (18)). This approximation is accurate only within specific ranges of SNR, BLER, and blocklength for the AWGN channel under consideration. Therefore, the normal approximation is not precise for small SNRs. However, small SNRs can be used in simulations of the early-detection scheme to evaluate its performance in environments where the noise is significant compared to the signal, allowing for realistic system modeling. This can help determine the optimal design, thus understanding the early-detection scheme’s performance at a low SNR can benefit system optimization.

Figure 6 shows the average latency as a function of the information block size *k* for a fixed code rate $R = 0.5$ under various channel conditions. It can be seen that, for an information block size of $k=1000$ bits, an average of $79\%$ of the symbol duration is required to meet an error rate of $10^{-5}$. Instead, $87\%$ of the symbol duration is needed for a given error rate of $10^{-2}$. In other words, as the error rate is low, the average symbol time needed decreases because the SNR to reach the required error is high. Furthermore, Fig. 6 also shows that as the information block size *k* grows, the symbol time needed to reach such an error probability increases. For example, for an information block size of $k \ge 10^{6}$ bits, the average latency needed to gets close to $100\%$ regardless of the targeted error performance. Thus, the use of the proposed EDS is more beneficial with smaller information block sizes.

**Fig. 7:**
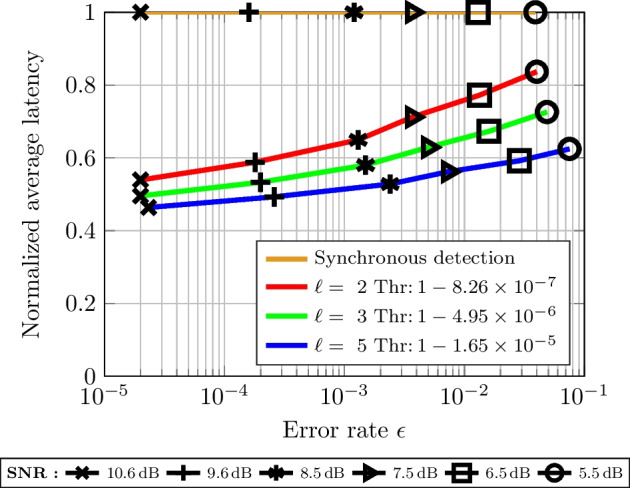
Normalized average latency of the proposed early-detection scheme compared to that of a synchronous-detection scheme under various channel conditions. The proposed detection scheme combines a list decoder with list size $\ell$ and a MSPRT, whose threshold (Thr) is indicated in the figure

Concerning the scheme described in Sect. 3.3.1, $M = 1024$ BPSK-modulated 10-bit messages are sent, where each symbol is transmitted in parallel AWGN channels. The list decoder provides the $\ell$ most probable codewords, by which the sequential test defined in (30) quickly makes the decision on the message if threshold in (31) is reached. The minimum size of the list must be two, and in this case, the sequential test becomes a SPRT. Figure 7 presents simulation results under various channel conditions of the EDS using MSPRT. From this figure, it can be seen that an EDS using sequential tests allows for significant latency reductions, especially if the SNR can be increased. For example, for a list size $\ell =3$, the proposed scheme can detect messages approximately $30\%$ to $50\%$ faster on average compared to synchronous detection, where the improvement is the most significant at higher SNR. It can also be seen that using a larger list size $\ell$ leads to a lower latency at the cost of a slight increase in error rate. Also, the threshold value slightly reduces as the list size grows.

As seen above, the proposed scheme uses the $\ell$-nearest distance between the received message and the *M* possible messages. However, such a selection of distances can be computationally prohibitive. Fortunately, many efficient decoding algorithms provide the $\ell$ most-probable codewords, such as list-decoding algorithms for Reed-Solomon [30] or polar codes [31].

**Fig. 8:**
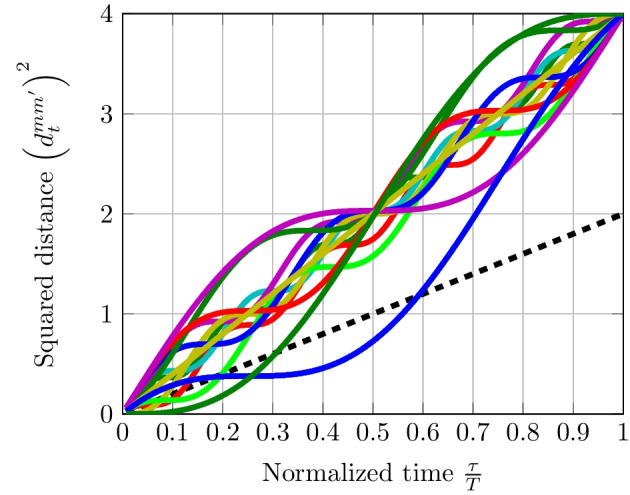
Distances over time between OFDM signals with 128 subcarriers and where codewords are mapped to a QPSK modulation. Number of different symbols in codewords $\#\mathcal {K} = 1$ represented by the black dashed curve; number of different symbols in codewords $\#\mathcal {K} = 2$ represented by colored curves

Concerning EDS with OFDM as discussed in Sect. 3.3.2, when the number of elements of the subset $\mathcal {K}$ denoted by $\#\mathcal {K}$ is equal to one, we can find from (36) that $\left( d_{t}^{mm'}\right) ^{2}$ is linear. However, when $\#\mathcal {K} = 2$, the distances over time grow linearly but a sinusoid of a frequency $(k_{1}-k_{2})/T$ has to be taken into account due to the overlapping dimension. Figure 8 shows the behavior of the squared distance over time. In $\mathcal {K}$, we have modified one single bit in each dimension. When $\#\mathcal {K}$ increases, there is a superposition of multiple sinusoids of a frequency $(k_{i}-k_{j})/T~\forall k_{i}$ $\ne k_{j} \in \mathcal {K}$ which tends to linearize distances over time. Hence, $\#\mathcal {K}\ge 2$ is equivalent to the use of a random coding scheme.

**Fig. 9:**
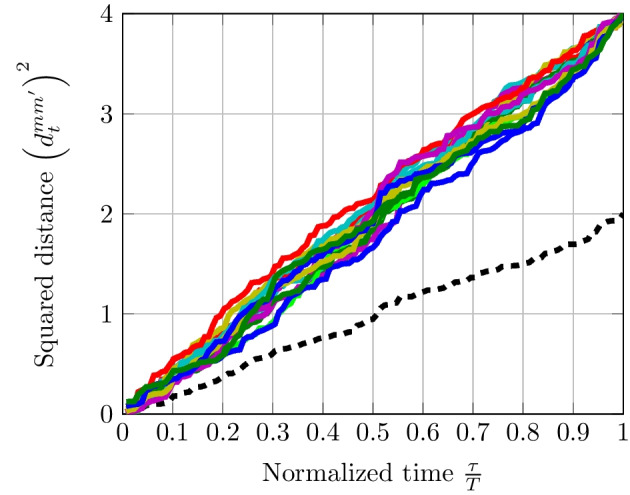
Distances over time between OFDM signals where codewords have been pre-coded by a Hadamard orthogonal matrix. Number of different symbols in codewords $\#\mathcal {K} = 1$ represented by the black dashed curve; number of different symbols in codewords $\#\mathcal {K} = 2$ represented by colored curves

By taking results obtained in Fig. 8, we apply a complex-valued $128 \times 128$ Hadamard orthogonal matrix $\textbf{H}$ to these codewords as indicated in (37) and (38). Figure 9 shows that $\left( d_{t}^{mm'}\right) ^{2}$ are approximately linear which could render the EDS efficient. These results show that there is evidence that latency can be reduced with pre-coded OFDM signaling by using EDS. Specifically, we prove in Fig. 9 that the EDS is feasible with OFDM signals once its distances have been linearized using random coding schemes and pre-coding orthogonal matrices.

Finally, we present the results for different SNRs and blocklengths of the EDS for the previously analyzed multi-hop system in Sect. 3.3.3. This latency comparison employs Theorem 3.12. To determine the latency reduction, we compare the results obtained with the EDS and the results obtained with synchronous detection for different $(n,M,\epsilon )$-codes. Then, we express the average latency in terms of normalized symbols.

**Fig. 10:**
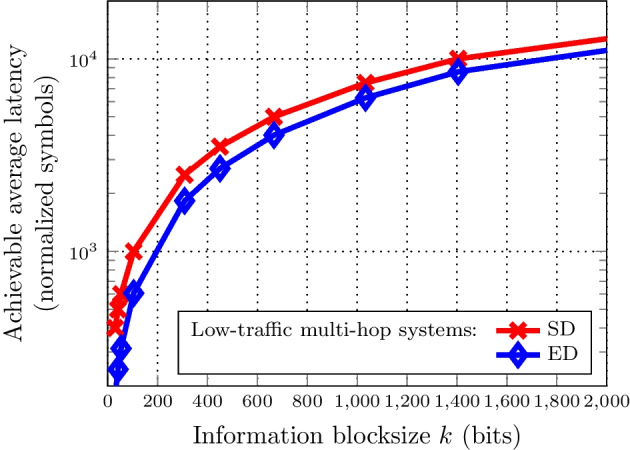
Optimal achievable average latency of low-traffic multi-hop systems using either synchronous detection (SD) or early detection (ED). The number of hops $h = 5$, the BLER $\epsilon = 10^{-5}$, and the signal-to-noise ratio (SNR) is 5 dB

Figure 10 shows the optimal achievable average latency for low-traffic multi-hop systems using either synchronous or early detection. The results are for 5 hops, a 5 dB SNR and a BLER of $\epsilon = 10^{-5}$. It can be seen that for such a multi-hop scheme, the use of an EDS leads to a lower latency in comparison to synchronous detection, regardless of the information blocksize. Additionally, it can be seen that, for both schemes, the latency increases in a similar fashion as the block size increases.

**Fig. 11:**
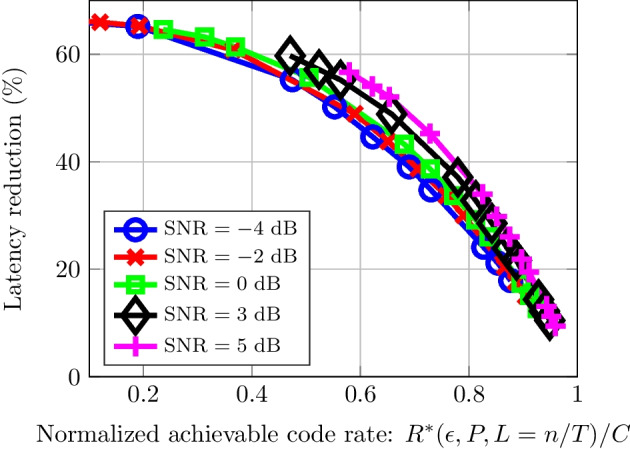
Latency reduction for normalized achievable code rates with various SNR link budgets. The number of hops $h = 5$, and the BLER $\epsilon = 10^{-5}$

Figure 11 shows the latency reduction achieved for various normalized achievable code rates and SNRs. The number of hops is $h = 4$, and the BLER is $\epsilon = 10^{-5}$. It can be seen that the latency reduction obtained with the proposed EDS decreases as the code rate tends to capacity. The tendencies are the same across all plotted SNRs. The most significant decrease in latency is present in low-rate codes with a reduction close to 65%. Figure 11 also shows a latency reduction greater than 40% over a wide range of code rates, especially at higher SNR per hop.

## Conclusion

In this work, we introduced new optimal low-latency communication strategies. Combined together, they form an early-detection scheme (EDS). By focusing on fixed-rate coding in the finite blocklength regime, the key ingredient of the proposed EDS is the use of sequential tests to make decisions before the end of the symbol duration. Furthermore, compared to conventional sequential detection techniques, our proposal does not necessitate feedback, thus avoiding additional latency.

In that regard, we derived the minimal achievable latency over the additive white Gaussian noise (AWGN) channel for both synchronous detection and our EDS. We also demonstrated that the EDS attains the optimal achievable latency by minimizing the time needed to make decisions. For example, we showed that messages with a blocklength of 500 symbols could be correctly received using only $63\%$ and $71\%$ of the symbol time while maintaining a block error rate of $10^{-5}$ for rates *R* of 0.5 and 0.95 bits per channel use, respectively.

Moreover, we developed a sequential test based on a multihypothesis sequential probability-ratio test (MSPRT). Results showed that, compared to synchronous detection, receivers could make decisions faster while maintaining the required reliability. Such scheme is effective if all symbols are simultaneously transmitted in parallel AWGN channels via orthogonal frequency-division multiplexing (OFDM)-like signals. However, it should be kept in mind that the proposed technique for OFDM signals is feasible if the message is pre-coded by random matrices, which guarantees no loss of orthogonality.

As our proposed EDS would allow relay nodes to start their retransmission earlier, we think that they are promising solutions for ultra-reliable and low-latency communications (URLLC) transmissions in multi-hop communications. Finally, as future work, it would be interesting to extend the proposed schemes to fading channels.

## Data Availability

Please contact the corresponding author at dobarragan@utpl.edu.ec

## Abbreviations

AWGN: Additive white Gaussian noise
AF: Amplify-and-forward
BPSK: Binary phase-shift keying
DF: Decode-and-forward
KL: Kullback–Leibler
MSPRT: Multihypothesis sequential probability-ratio test
OFDM: Orthogonal frequency-division multiplexing
QPSK: Quadrature phase-shift keying
SPRT: Sequential probability-ratio test
SNR: Signal-to-noise ratio
eMBB: Enhanced mobile broadband
mMTC: Massive machine-type communication
URLLC: Ultra-reliable and low-latency communications
MSPRT: Multihypothesis sequential probability ratio test
IBL: Infinite blocklength FBL finite blocklength
DAF: Decode-amplify-and-forward
DEF: Decode-estimate-and-forward
DmF: Demodulate-and-forward
EF: Estimate-and-forward
MTC: Machine type communications
IoT: The Internet-of-Things
BLER: Block error rate
5 G: Fifth-generation cellular network technology
i.i.d.: Independent and identically distributed
LLR: Log-likelihood ratio
EDS: Early-detection scheme
SD: Synchronous detection.

## Footnotes

1 This paper was presented in part at ISWCS 2019 [13]

2 For a given SNR, the channel dispersion of a complex-valued AWGN channel is double that of a real-valued AWGN channel.

3 It is common to assume that the receiver knows the channel gain because it can be tracked and estimated using a training sequence.

## References

[1] Wunder G, Jung P, Kasparick M, Wild T, Schaich F, Chen Y, Brink ST, Gaspar I, Michailow N, Festag A, Mendes L, Cassiau N, Ktenas D, Dryjanski M, Pietrzyk S, Eged B, Vago P, Wiedmann F (2014). 5GNOW: non-orthogonal, asynchronous waveforms for future mobile applications. IEEE Commun. Mag..

[2] Bennis M, Debbah M, Poor HV (2018). Ultrareliable and low-latency wireless communication: tail, risk, and scale. Proc. IEEE.

[3] J. Östman, G. Durisi, E.G. Stroem, J. Li, H. Sahlin, G. Liva, Low-latency ultra-reliable 5G communications: finite-blocklength bounds and coding schemes, in *Int. ITG Conf. on Syst., Commun. Coding (SCC)*, pp. 1–6 (2017)

[4] H. Kim, Ultra-reliable and low latency communication systems. in *Design and optimization for 5G wireless communications*, 1st edn., pp. 303–342. IEEE, West Sussex (2020). 10.1002/9781119494492.ch8

[5] Chen H, Abbas R, Cheng P, Shirvanimoghaddam M, Hardjawana W, Bao W, Li Y, Vucetic B (2018). Ultra-reliable low latency cellular networks: use cases, challenges and approaches. IEEE Commun. Mag..

[6] Feng D, Lai L, Luo J, Zhong Y, Zheng C, Ying K (2021). Ultra-reliable and low-latency communications: applications, opportunities and challenges. Sci. China Inf. Sci..

[7] Durisi G, Koch T, Popovski P (2016). Toward massive, ultrareliable, and low-latency wireless communication with short packets. Proc. IEEE.

[8] Polyanskiy Y, Poor HV, Verdu S (2010). Channel coding rate in the finite blocklength regime. IEEE Trans. Inf. Theory.

[9] Dragalin VP, Tartakovsky AG, Veeravalli VV (1999). Multihypothesis sequential probability ratio tests. I. Asymptotic optimality. IEEE Trans. Inf. Theory.

[10] Baum CW, Veeravalli VV (1994). A sequential procedure for multihypothesis testing. IEEE Trans. Inf. Theory.

[11] Siegmund D (1985). Sequential Analysis. Tests and Confidence Intervals.

[12] Kazovsky LG (1985). Sequential detection versus conventional detection: a comparative study. Signal Process..

[13] D. Barragán, M. Au, G. Gagnon, F. Gagnon, P. Giard, Early detection for optimal-latency communications in multi-hop links. in *Int. Symp. on Wireless Commun. Syst. (ISWCS)*, pp. 389–394 (2019). 10.1109/ISWCS.2019.8877294

[14] Shannon CE (1948). A mathematical theory of communication. Bell Syst. Tech. J..

[15] Wald A (1945). Sequential tests of statistical hypotheses. Ann. Math. Statist..

[16] Mengali U (1973). An alternative signaling scheme for sequential decision feedback. Proc. IEEE.

[17] Shirvanimoghaddam M, Mohammadi MS, Abbas R, Minja A, Yue C, Matuz B, Han G, Lin Z, Liu W, Li Y, Johnson S, Vucetic B (2019). Short block-length codes for ultra-reliable low latency communications. IEEE Commun. Mag..

[18] Viterbi AJ (1965). The effect of sequential decision feedback on communication over the Gaussian channel. Inform. Contr..

[19] Kramer AJ (1967). Use of orthogonal signaling in sequential decision feedback. Inform. Contr..

[20] Proakis J, Salehi M (2008). Digital communications.

[21] Zaidi A, Athley F, Medbo J, Gustavsson U, Durisi G, Chen X (2018). 5G physical layer: principles, models and technology components.

[22] Durisi G, Koch T, Östman J, Polyanskiy Y, Yang W (2016). Short-packet communications over multiple-antenna Rayleigh-Fading channels. IEEE Trans. Commun..

[23] Tan VYF, Tomamichel M (2015). The third-order term in the normal approximation for the AWGN channel. IEEE Trans. Inf. Theory.

[24] Johnson CR, Sethares WA, Klein AG (2011). Software receiver design: build your own digital communications system in five easy steps.

[25] P. Mary, J. Gorce, A. Unsal, H.V. Poor, Finite blocklength information theory: What is the practical impact on wireless communications? in *2016 IEEE Globecom Workshops (GC Wkshps)*, pp. 1–6 (2016)

[26] López OLA, Alves H, Souza RD, Latva-Aho M (2020). Finite blocklength error probability distribution for designing ultra reliable low latency systems. IEEE Access.

[27] Dragalin VP, Tartakovsky AG, Veeravalli VV (2000). Multihypothesis sequential probability ratio tests II. Accurate asymptotic expansions for the expected sample size.. IEEE Trans. Inf. Theory.

[28] Veeravalli VV, Baum CW (1995). Asymptotic efficiency of a sequential multihypothesis test. IEEE Trans. Inf. Theory.

[29] Poor HV, Hadjiliadis O (2008). Quickest Detection, 1.

[30] El-Khamy M, McEliece RJ (2006). Iterative algebraic soft-decision list decoding of Reed-Solomon codes. IEEE J. Sel. Areas Commun..

[31] Tal I, Vardy A (2015). List decoding of polar codes. IEEE Trans. Inf. Theory.

